# Effect of New Eco-Polyols Based on PLA Waste on the Basic Properties of Rigid Polyurethane and Polyurethane/Polyisocyanurate Foams

**DOI:** 10.3390/ijms22168981

**Published:** 2021-08-20

**Authors:** Marcin Borowicz, Marek Isbrandt, Joanna Paciorek-Sadowska

**Affiliations:** Department of Chemistry and Technology of Polyurethanes, Institute of Materials Engineering, Kazimierz Wielki University, 30 Chodkiewicza Street, 85-064 Bydgoszcz, Poland; m.isbrandt@ukw.edu.pl (M.I.); sadowska@ukw.edu.pl (J.P.-S.)

**Keywords:** PLA waste, chemical recycling, polyol, polyurethane foam, polyurethane/polyisocyanurate foam, foam properties

## Abstract

The aim of the presented research was to obtain two new eco-polyols based on waste polylactide (PLA) and to check the effect on the properties of rigid polyurethane (RPU) foams and, based on these, rigid polyurethane/polyisocyanurate (RPU/PIR) foams. The synthesis of eco-polyols was based on the transesterification reaction of melted PLA with diethylene glycol in the presence of an organometallic catalyst. Properties of the obtained eco-polyols were examined for their potential as raw materials for synthesis of rigid polyurethane and polyisocyanurate foams, i.e., hydroxyl value, acid value, density, viscosity, pH, water content. Spectroscopic studies (FTIR, ^1^H NMR and ^13^C NMR) were also carried out. Results of these tests confirmed the assumed chemical structure of the new polyols. RPU and RPU/PIR foam formulations were developed based on the obtained analytical results. Partial replacement of petrochemical polyol by eco-polyols in RPU and RPU/PIR foams decreased the value of apparent density, compressive strength, brittleness and water absorption. Moreover, all foams modified by eco-polyols showed higher resistance to aging. All RPU/PIR foams and most PRU foams modified by eco-polyols from waste PLA had better functional properties than the reference foams based on petrochemical polyol.

## 1. Introduction

Nowadays, the polyurethane (PU) materials industry is developing at a very fast rate. Statistical data show that the world production in this sector in 2019 amounted to 22.5 million tons. It was an increase of about 3.5% in comparison with the previous year. It is estimated that in 2025 the global production of polyurethanes will grow to 25 million tons [1,2,3,4]. It is related to the rapid development of the furniture, construction (mainly in thermal insulation), automotive and footwear industries. Undoubtedly, the polyurethane industry is gaining importance among all polymeric materials due to the massive demand for flexible foams, rigid foams, coatings, elastomers and adhesives [5,6,7,8,9,10,11]. A very desirable feature of these materials are their properties, which can be modified depending on what is needed by changing the type of raw materials, their quantity ratio and the density of the final product in the range from 12 kg/m^3^ to 1000 kg/m^3^. None of the previously known plastics have such a wide range of modifications [12].

Along with the increasing demand for polyurethane materials, the demand on the polyol raw material market is also increasing. Currently, there are three main groups of polyols. These include polyether polyols, polyester polyols and other polyols (e.g., bio-polyols, eco-polyols, glycolysates, etc.). In 2016, more than 75% of the total production of polyols used in the production of polyurethane materials were polyether polyols (approximately 7.2 million tons). The remaining 25% (2.4 million tons) were polyester polyols. The polyols market is developing very dynamically, noting the average increase of approximately 4% [1,9,13,14,15,16].

In the last decade, global polymer production has increased significantly and reached 359 million tons in 2018. Comparing these statistical data with 2017, an upward trend of 3.2% is observed. This proves a large increase in the demand for polymeric materials. On the one hand, it is a positive aspect that proves the development of the world economy. On the other hand, we must ask ourselves: what about the increasing amount of waste that will have to be dealt with in the near future [17]?

One of the main goals of European legislation is the implementation of sustainable waste management and reasonable management of natural resources. Its main tasks are promoting the idea of a “recycling society” and promoting a recycling hierarchy based on the slogan “Reduce, Reuse, Recycle, Recover”. It is aimed at reducing waste generation and using it as a resource through material, chemical and biological recycling or energy recovery [18,19,20]. The importance of these methods of waste management is growing every year, and the methods of recycling polymeric materials are still being improved [21]. New legal regulations on environmental protection also pose challenges for scientists related to the search for new technologies for the management of plastic waste [22,23]. They mainly focus on “green” polymers (biodegradable polymers) [24] that degrade under appropriate environmental conditions [25].

In the 1970s, humanity exceeded the safe limit of the exploitation of earth. This means that global production uses more natural resources than nature can restore. It is possible to cross this limit, but for a very short period of time. This means, for example, that we can emit more carbon dioxide into the atmosphere than forests can process or catch more fish from the oceans than they can reproduce. However, it should be remembered that the overexploitation of our planet will lead to the destruction of the environment and, consequently, of people [26].

The circular economy model is the answer to the current threats leading to environmental degradation. It focuses mainly on how to reduce the destructive impact of production on the environment, and in particular to reduce waste generation. In order to achieve the assumed goals, it is necessary to undertake systemic and integrated actions [27]. The circular economy has now become the overarching goal of the EU’s economic policy. At the end of 2015, the European Commission published a number of legal acts related to the circular economy policy for the Member States. These regulations cover the entire life cycle of a product, from production (product design), through consumption, to waste management in the secondary raw materials industry. With its actions, the EU plans to achieve by 2030 the value of recycling of municipal waste at 65% and packaging waste at the value of 75% [28]. According to the European Commission, the definition of the concept of a circular economy is an economy in which the value of products and materials is maintained for as long as possible, and the production of waste is kept to a minimum. It also enables the optimal reuse, regeneration and recycling of the obtained products [29]. The circular economy is closely related to the extension of the “life cycles” of products [22,27,30].

In the polyurethane industry, the implementation of a circular economy combined with the principles of “green chemistry” is currently being observed. This is evident by, e.g., elimination of petrochemical polyols in favor of plant-derived polyols [31,32,33,34,35] or those obtained from chemical recycling [36,37] and the addition of waste fillers into polyurethane formulation [38,39,40,41].

Currently, several types of biodegradable plastics are produced. The most popular of this plastics group is polylactide (PLA). It is an alternative to traditional synthetic materials obtained through the processing of fossil fuels because it has properties similar to them. Moreover, it is obtained from renewable sources, including corn and rice [21,42]. Polylactide is the material of the future, which is currently visible in the industry. The global production of this biopolymer in 2017 amounted to 0.2 million tons, which is approximately 10.3% of all biodegradable polymers [43]. This polymer is widely used in the production of bottles, disposable cutlery and trays, foil, packaging, as well as in 3D printing technology [44]. The use of this material on a mass scale generates a large amount of biodegradable waste. Biodegradation as a chemical process has advantages and disadvantages. A positive aspect is the fact that these polymers decompose into carbon dioxide or methane, biomass and water under composting conditions. On the other hand, the negative impact of this process is that the benefits, such as recovery of the monomer and its reuse in the polymerization process, are not achieved. The situation is different when chemical recycling of PLA is used [21]. In 2016, for the first time in Europe, the amount of recycled PLA waste exceeded landfilling [17]. This proves that new technologies for the processing of polylactide waste into finished products are being developed. Therefore, the authors showed a method that perfectly fits into the doctrine of the circular economy, i.e., chemical recycling of polylactide waste into polyol raw material, and research on its influence on the properties of new polyurethane materials. This type of research has not been conducted in any research center before. This method is very innovative and, above all, waste-free, because it enables the processing of potentially biodegradable plastic and its use as a full-value product. This is important because it also enables the obtaining of a positive economic balance.

The aim of this research was to use the glycolysis reaction of PLA waste as an alternative method of recycling this material and to check the usefulness of the obtained glycolysis products for the synthesis of rigid polyurethane (RPU) foams and rigid polyurethane/polyisocyanurate (RPU/PIR) foams.

## 2. Results and Discussion

### 2.1. Properties of New Eco-Polyols

#### 2.1.1. Physicochemical and Analytical Tests

**Figure 1:**
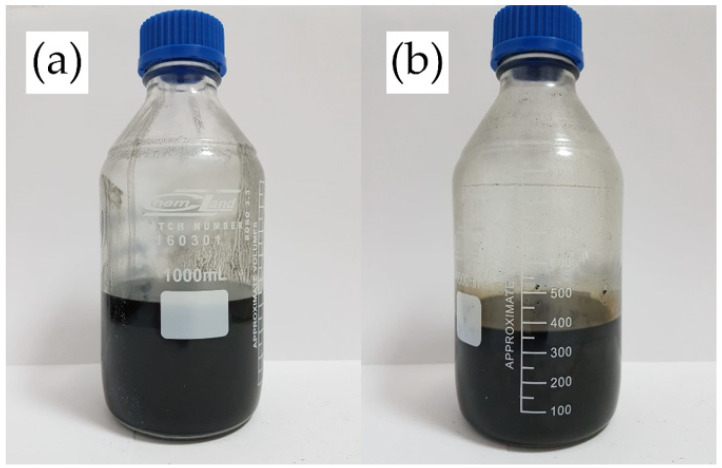
Appearance of eco-polyols: (**a**) PLA0.4DEG; (**b**) PLA0.3DEG.

Two eco-polyols were obtained as a result of the transesterification reaction of PLA waste with diethylene glycol in a weight ratio of 1:0.4 and 1:0.3 (PLA:DEG). Contrary to the PLA waste, the two polyol raw materials were liquids at room temperature. The basic physicochemical properties of the new eco-polyols are presented in Table 1. The appearance of the obtained polyols is shown in Figure 1.

The properties of eco-polyols based on waste PLA were different depending on the amount of diethylene glycol used. The only parameter of polyol raw materials which was independent of reagents ratio was color. It depended on the color of the polylactide waste used in the synthesis. Using waste, it is not possible to clearly indicate the color of the final product after the reaction. The color of polyols is strongly dependent on the pigments in the processed waste. During the reaction, all pigments from PLA are mixed to give the final color of the eco-polyol. Using even pure, unmodified PLA does not guarantee the obtaining of a colorless polyol [22]. The synthesized eco-polyols did not have any smell. This suggests that the reaction did not produce any volatile low molecular weight products. The lack of smell also meant that all of the diethylene glycol had completely reacted with the PLA waste. The density of the PLA0.4DEG and PLA0.3DEG eco-polyols was the same and was 1.24 g/cm^3^. The density of eco-polyols was the same as the density of available commercially polylactide used for production of 3D printing filament [45]. The main difference was noted in the viscosity of the obtained compounds. The viscosity of PLA0.3DEG was almost four times higher than that of PLA0.4DEG. This was due to the lower amount of transesterification agent used for the reaction with PLA waste. The increase in viscosity of the PLA0.3DEG polyol in comparison with PLA0.4DEG was mainly due to the change in the chain length of the transesterified PLA. In the case of the PLA:DEG mass ratio of 1:0.4, products with shorter chains were obtained than in the case of the reactant ratio of 1:0.3. A 25% reduction in the amount of DEG in the reaction system resulted in longer chain products, which directly resulted in a four-fold increase in viscosity. The increase in viscosity is undesirable from an economic point of view. However, both eco-polyols met industry standards which assume that the viscosity of industrial polyols should be in the range of 500–10,000 mPa·s [9,11,46]. The pH of both eco-polyols was 6.6. This meant that the obtained products were slightly acidic. This was due to the presence of a small amount of carboxyl groups at the end of the chains that had not reacted with DEG.

**Figure 2:**
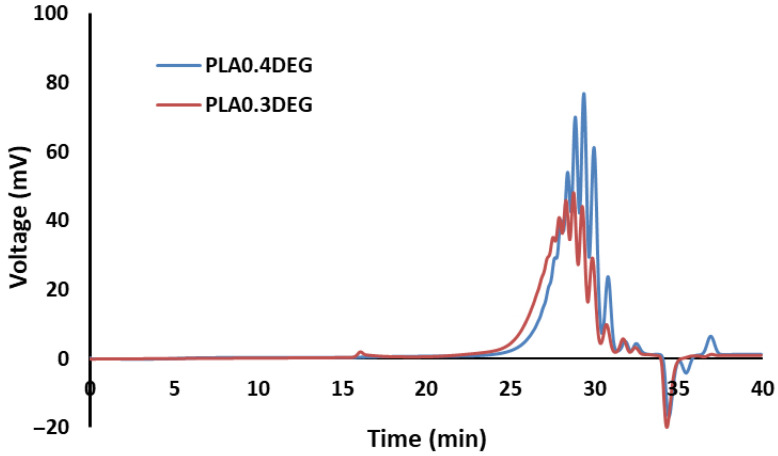
GPC chromatograms of eco-polyols based on waste PLA.

A number of analytical tests were carried out, such as determination of the hydroxyl number (HV), acid number (AV), water content (%_H2O_), and elemental composition or molecular weight, in order to determine the suitability of the obtained eco-polyols for the synthesis of polyurethane materials and for a better understanding of their properties. The obtained results are presented in Table 2, Table 3 and Table 4 and in Figure 2.

One of the most important parameters determining the usefulness of polyhydric alcohols for the synthesis of polyurethane materials is the hydroxyl value (HV). It directly affects the type of obtained PU material, e.g., rigid foam, flexible foam, elastomer, etc. It also affects the properties of the obtained material [9,47]. In the case of PLA0.4DEG and PLA0.3DEG eco-polyols, it was noted that HV was 261.64 and 209.87 mg KOH/g, respectively. The higher content of DEG in relation to PLA waste during the synthesis resulted in the formation of shorter oligomer chains. Thus, it caused the release of more hydroxyl groups during the transesterification of the polylactide. This directly influenced the higher HV value for PLA0.4DEG eco-polyol in comparison with PLA0.3DEG eco-polyol. The change in the ratio of PLA:DEG reagents from 1:0.4 to 1:0.3 resulted in an almost 20% decrease in the value of this parameter. The acid value (AV) of the obtained eco-polyols was approximately 2 mg KOH/g. AV is the number of free carboxyl groups present in the obtained compound. The presence of free COOH groups is natural in this case. The PLA transesterification reaction was aimed at the replacement of the OH group from the lactic acid monomer with the OH group derived from DEG, and not the esterification of the end carboxyl group [48]. The water content of the obtained eco-polyols was 0.1 wt.% for PLA0.4DEG and 0.25 wt.% for PLA0.3DEG, respectively. It is a relatively small amount of water that did not need to be removed by vacuum distillation. In particular, for ecological reasons, polyurethane materials were to be foamed with carbon dioxide obtained by reacting water with an excess of isocyanate raw material. Due to the fact that the tranesterification reaction did not result in the formation of low molecular weight products (such as water), it had to be introduced into the system along with DEG, or it could have been absorbed from the air during cooling or in the filtration process (both operations were performed in open vessels).

The synthesized PLA0.4DEG and PLA0.3DEG eco-polyols were analyzed for their elemental composition. The study of the share of individual elements in the compound is very important because it allows us to calculate the theoretical oxygen demand (TOD). This parameter is important when calculating the degree of biodegradation of the tested material [49]. The results of the elemental analysis of the obtained eco-polyols based on PLA waste are presented in Table 3.

On the basis of the obtained results of elemental analysis, it was found that the change in the amount of DEG used in the reaction did not have a significant effect on the changes in the share of carbon, hydrogen and oxygen in the eco-polyol molecule.

The obtained PLA transesterification products were also subjected to a gel permeation chromatography (GPC) test. In this study, the number average molecular weight (*M_n_*), the weight average molecular weight (*M_w_*), and the dispersity (D) were determined. The GPC chromatograms of both eco-polyols are shown in Figure 2. The results of their analysis are presented in Table 4. Moreover, the functionality of the obtained eco-polyols was calculated on the basis of the number average molecular weight and the determined hydroxyl value (Equation (1)):

(1) $$f=\frac{M_{n}\cdot HV}{56,100}$$

where: *f*—functionality (-), *M_n_*—the number average molecular weight (g/mol), *HV*—hydroxyl value (mg KOH/g).

The results presented in Table 4 were obtained on the basis of averaging the results obtained for individual peaks of the chromatogram in Figure 2. The GPC analysis of eco-polyols confirmed that there was an increase in the number-average molecular weight and the weight-average molecular weight when the amount of DEG in the reaction mixture decreased. This was due to the obtaining of longer chains of transesterified PLA with a lower amount of diethylene glycol [22]. Peaks with less than 0 mV were also noted in the chromatograms. This was related to the presence of small amounts of water in the tested eco-polyols, which could be introduced into eco-polyols at the synthesis stage (along with DEG) or absorbed from the air at the stage of product separation after synthesis (in the process of cooling or catalyst filtration). An important parameter of the obtained compounds is their dispersity (D). A high value of this parameter can significantly reduce or exclude the use of these raw materials in the production of polyurethane materials. Polyurethane materials obtained from polyols with low dispersity (D < 2) are characterized by an ordered structure and have favorable properties [50,51]. The dispersity of the obtained eco-polyols was 1.18 for PLA0.4DEG and 1.27 for PLA0.3DEG, respectively. Low polymolecularity is very advantageous because eco-polyols are to be used as raw materials for the synthesis of polyurethane foams. Polyols with molecular weight below 1000 g/mol and high functionality are most often used for the synthesis of RPU foams. The use of such polyols promotes the formation of highly cross-linked, rigid structures [52]. The functionality of the obtained glycolysates was 1.59 for PLA0.4DEG and 1.55 for PLA0.3DEG, respectively. These values were lower than expected. Theoretically, the functionality of one eco-polyol molecule should be 2 because it has one hydroxyl group derived from the lactic acid monomer and one hydroxyl group derived from the DEG molecule. The lower functionality resulted from the presence of chains in which a carboxyl group (end group) was also released as a result of the transesterification reaction. The presence of chains terminated with a free carboxyl group was also confirmed by the acid values (approx. 2 mg KOH/g).

#### 2.1.2. Spectroscopy Tests

During the synthesis of new polyol raw materials, it is necessary to confirm their chemical structure. For this purpose, three spectroscopic tests: FTIR, ^1^H NMR and ^13^C NMR, were performed. The obtained spectra were interpreted, among others, on the basis of spectroscopic spectra database—Spectral Database for Organic Compounds (SDBS) [53].

**Figure 3:**
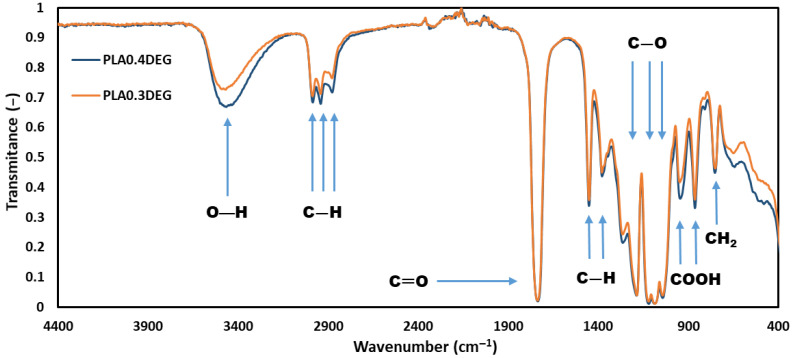
FTIR spectra of new eco-polyols.

The study of Fourier transform infrared spectroscopy (FTIR) was aimed at confirming the presence of characteristic functional groups in the new eco-polyols. The obtained FTIR spectra for PLA0.4DEG and PLA0.3DEG are shown in Figure 3.

Analysis of FTIR spectra of PLA-based polyols (Figure 3) showed that there were characteristic bonds of the structure of lactic acid esters. The spectra of eco-polyols showed high band intensity at 3400 cm^−1^, which indicated the presence of O–H bonds in the hydroxyl groups. Bands at 2880–3000 cm^−1^ (stretching) and 1360–1460 cm^−1^ (deformational) belonged to the C–H bond of the –CH_2_– and –CH_3_ group in lactic acid monomers and in DEG; bands at 1640–1760 cm^−1^ (stretching) belonged to the C=O bond of the ester group between lactic acid monomers or lactic acid and glycerol; bands at 1050–1270 cm^−1^ (stretching) belonged to the C-O bond of the ester group; bands at 870–930 cm^−1^ belonged to the free carboxyl group at the end of the chain of PLA and the pendulum vibrations of the CH_2_ group in 725 cm^−1^ [21,22,54,55,56]. Both spectra were practically identical. The only visible difference was the intensity of the transmission band at 3400 cm^−1^ belonging to the O–H bonds. For PLA0.4DEG eco-polyol it was clearly more intense than for PLA0.3DEG eco-polyol. This was due to the presence of a higher amount of free hydroxyl groups, which was also confirmed by the determination of the hydroxyl value of both eco-polyols [57].

**Figure 4:**
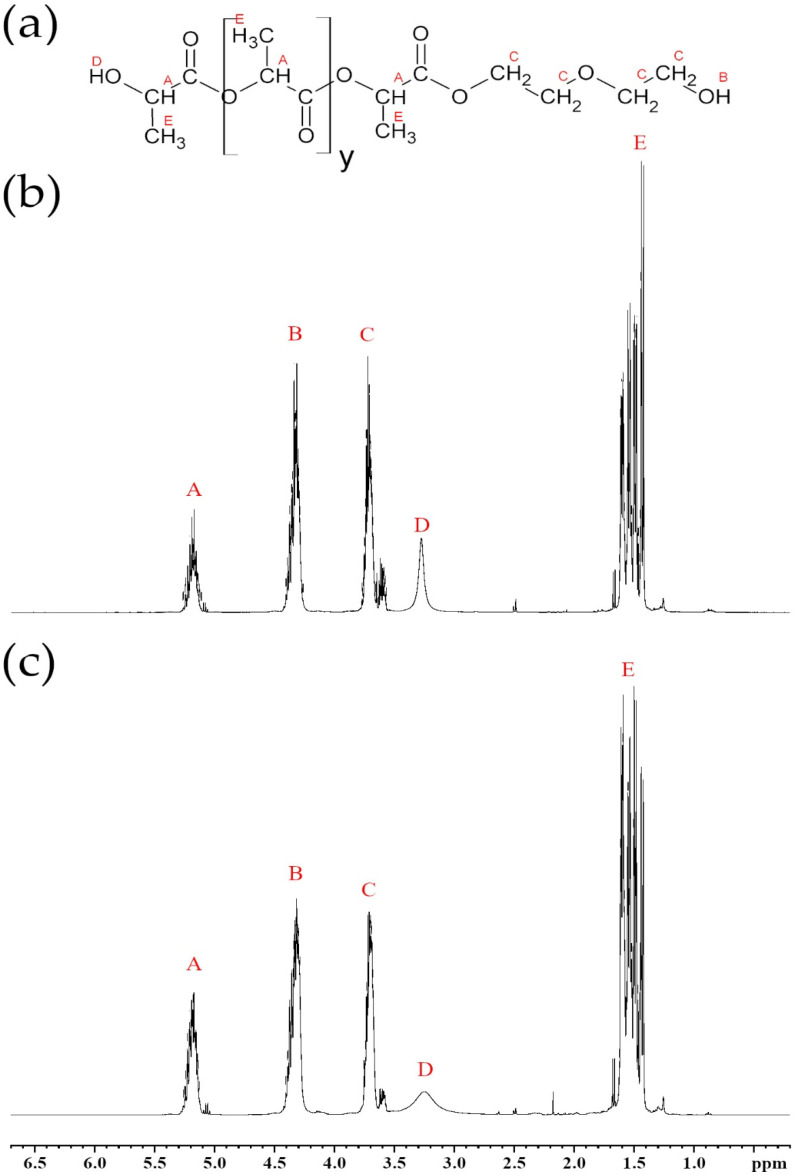
^1^H NMR spectra of new eco-polyols: (**a**) schematic structure of eco-polyols, (**b**) PLA0.4DEG spectrum, (**c**) PLA0.3DEG spectrum.

**Figure 5:**
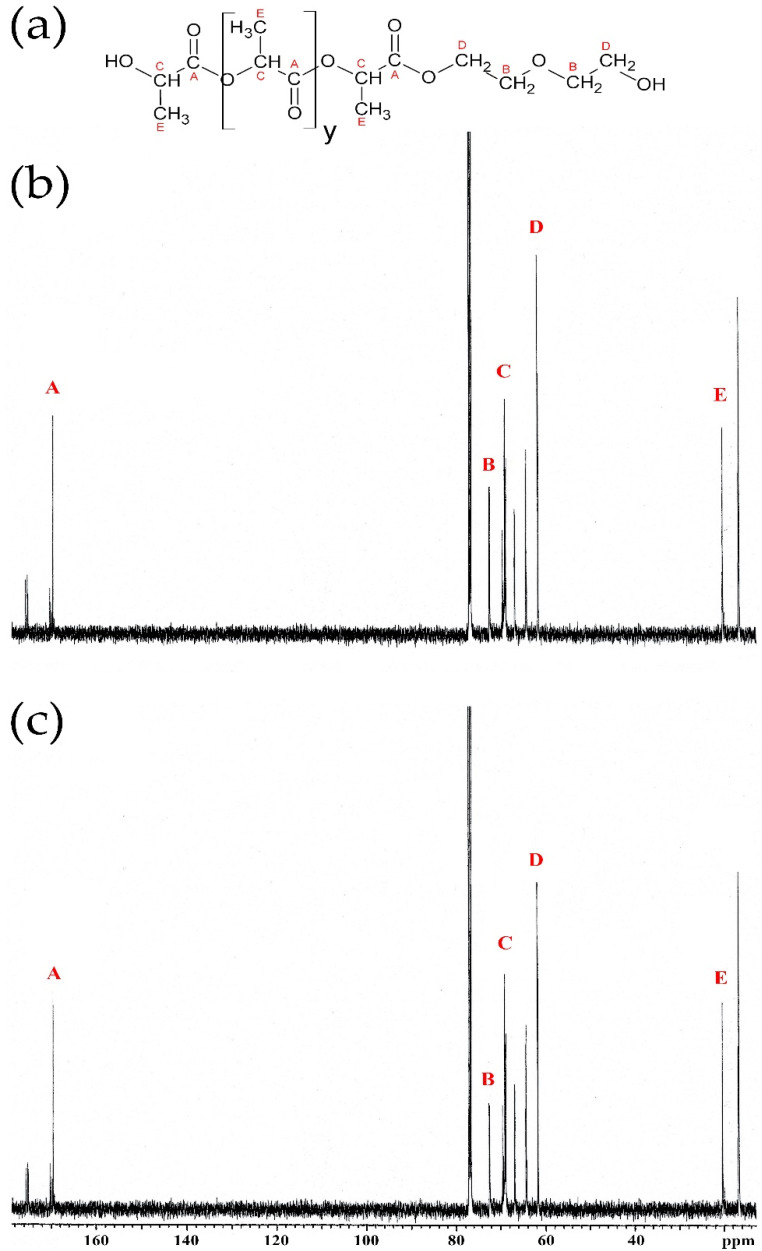
^13^C NMR spectra of new eco-polyols: (**a**) schematic structure of eco-polyols, (**b**) PLA0.4DEG spectrum, (**c**) PLA0.3DEG spectrum.

Analysis of eco-polyols in proton (^1^H NMR) and carbon (^13^C NMR) nuclear magnetic resonance spectroscopy confirmed the expected chemical structure. The ^1^H and ^13^C NMR spectra are presented in Figure 4 and Figure 5, respectively.

^1^H NMR spectra analysis of polyhydric alcohols showed characteristic chemical shifts for 5.15–5.30 ppm protons of α-CH groups to the ester group: (A) 4.25–4.40 ppm protons of hydroxyl groups at the end of DEG chains; (B) 3.60–3.80 ppm protons of α-CH_2_ groups to the alcoxyl group from glycol; (C) 3.25–3.40 ppm protons of hydroxyl group from lactic acid monomers; and (D) 1.40–1.60 ppm protons of methyl group from lactic acid monomers (E) [21,22,58,59,60]. The ^1^H NMR spectra of the eco-polyols based on PLA waste were identical. They differed slightly in the intensity of chemical shifts. This was due to the presence of PLA oligomer chains of different lengths (longer in PLA0.3DEG, shorter in PLA0.4DEG). In PLA0.4DEG, the peak of the proton chemical shift of the OH groups derived from DEG is higher than in PLA0.3DEG because more glycol was used in its synthesis.

^13^C NMR spectrum analysis showed characteristic chemical shifts for 169.50 ppm carbons of the carbonyl groups: (A) 72.50 ppm carbons of α-CH_2_ groups to the alcoxyl group; (B) 68.50–69.30 ppm carbons of α-CH_2_ groups to the ester group; (C) 61.50–61.70 ppm carbons of β-CH_2_ groups to the alcoxyl group; and (D) 16.60–20.40 ppm carbons of methyl groups (E) [21,22,61,62,63]. The 13C NMR spectra of PLA0.4DEG and PLA0.3DEG were practically identical. They only differed in the intensity of some chemical shift peaks, e.g., carbons of α-CH_2_. This was due to the different ratio of DEG to PLA during the synthesis of eco-polyols.

FTIR, ^1^H NMR and ^13^C NMR spectroscopic analyses confirmed the assumed chemical structure of the synthesized eco-polyols.

#### 2.1.3. Susceptibility to Biodegradation

**Figure 6:**
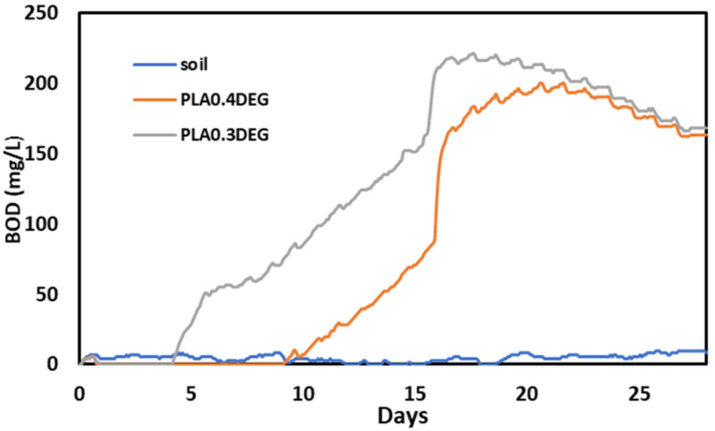
Results of biochemical oxygen demand of eco-polyols during 28 days in soil.

A growing problem is the rapid increase in the consumption of polymers, and thus also the increase in waste generation. PLA is one of the biodegradable polymers due to its natural origin. It is mainly obtained from corn or rice. Therefore, it is assumed that products based on it will also be susceptible to biodegradation [64,65]. The changes in biochemical oxygen demand (BOD) during 28 days were tested using the OxiTop Control S6 apparatus to check the biodegradability of products based on it. Liquid samples of eco-polyols were introduced into the system containing the previously prepared soil solution. The course of BOD changes during the 28 days of measurement is shown in Figure 6.

Final BOD was noted for both samples after 28 days. The mass shares of carbon, hydrogen and oxygen were calculated based on the results of elemental analysis (Table 3). The obtained values were used to calculate the theoretical oxygen demand (TOD). With the measured BOD_28_ and the calculated TOD, the degree of biodegradation (D_t_) of the obtained PLA0.4DEG and PLA0.3DEG eco-polyols was determined. The results are presented in Table 5.

The measured BOD_28_ value was higher than the calculated TOD value for both eco-polyols. This meant that the analyzed compounds influenced the development of aerobic microorganisms contained in the soil. The standard states that when the BOD_28_ value is higher than the TOD value, it should be considered that the tested compounds are completely biodegradable in the soil environment [49,66]. However, it should be remembered that this test is a simplified analysis of biodegradability, because it only refers to environmental parameters and does not take into account the microbiological aspects of the test environment.

### 2.2. Selected Properties of RPU and RPU/PIR Foams

#### 2.2.1. Foaming Process

The processing times were measured by an electronic stopwatch during the synthesis of RPU and RPU/PIR foams. Cream, string gel, tack free and free rise times were marked with this method. The results of RPU foam processing times are presented in Table 6.

Processing times of RPU foams based on PLA eco-polyols showed a tendency to elongation. A slight elongation of the cream time and a significant elongation of the remaining processing times were noted after using PLA0.4DEG eco-polyol. With the highest content of this eco-polyol (0.5 Eq), string gel, tack free and free rise times were almost twice as long as those of the reference foam (Ref. PUR). In the case of the PLA0.3DEG eco-polyol, a significant elongation of all processing times was noted. Using 0.5 Eq of this polyol, string gel, tack free and free rise times were even six times longer than in the case of Ref. PUR foam. The obtained results suggested that PLA0.4DEG and PLA0.3DEG eco-polyols had lower reactivity than petrochemical polyol (Rokopol RF-551). This was also confirmed by the lower hydroxyl value and lower functionality of PLA-based polyols in comparison with the reference polyol [47].

In the case of RPU/PIR foam synthesis, a different dependence was noted than in the case of RPU foam synthesis. All processing times of the foams modified by PLA0.4DEG and PLA0.3DEG eco-polyols were the same as processing times of reference foam (Ref. PIR). They were, respectively: cream time—7 s, string gel time—15 s, tack free time—18 s, and free rise time—27 s. The reason for this was the presence of a second catalyst (33% solution of anhydrous potassium acetate in DEG), which accelerated the trimerization reaction of NCO groups to the isocyanurate ring. Very often organometallic catalysts also affect the reaction between OH and NCO groups. Moreover, additional heat is also generated as a result of the trimerization reaction. It supports the action of the amine catalyst, e.g., 1,4-diazabicyclo[2,2,2]octane [9,67,68].

#### 2.2.2. Physicomechanical Properties of New Polyurethane Materials

**Figure 7:**
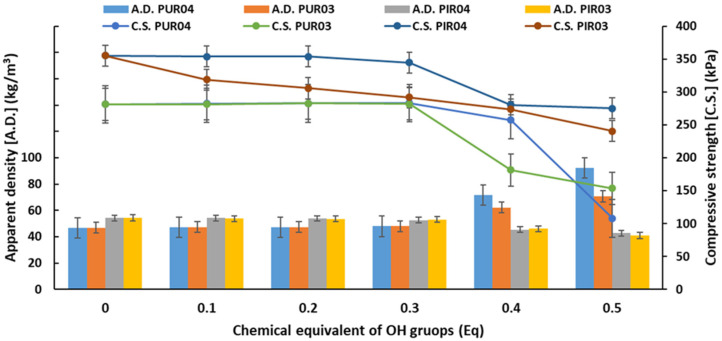
Dependence of apparent density (A.D.) and compressive strength (C.S.) on the content of eco-polyols based on waste PLA.

An important parameter of foamed polyurethane materials is their apparent density. It affects, among others, the mechanical strength of the obtained foams. The dependence between the apparent density, compressive strength of RPU and RPU/PIR foams and the content of eco-polyols is shown in Figure 7.

Rigid polyurethane foams are cross-linked materials which are characterized by a low apparent density. This parameter is usually in the range of 28 to 70 kg/m^3^. Due to the low apparent density, rigid polyurethane foams are used primarily for insulation purposes or for light construction and insulation materials [16,69]. In the case of synthesis of RPU foams based on eco-polyols, a significant impact of these raw materials on the value of apparent density was noted. Both after using PLA0.4DEG and PLA0.3DEG eco-polyols, this parameter increased with the increase in the number of eco-polyols from 46.76 kg/m^3^ for the reference foam (Ref. PUR) to 92.36 kg/m^3^ for PUR04.5 (0.5 Eq of PLA0.4DEG eco-polyol) and 70.7 kg/m^3^ for PUR03.5 (0.5 Eq of PLA0.3DEG eco-polyol), respectively. It was noted in both cases that a significant increase in apparent density occurred only after introducing at least 0.4 Eq of eco-polyols. The values of this parameter were at the level of the reference foam up to 0.3 Eq of eco-polyols. A similar dependence was also noted for the compressive strength of RPU foams based on PLA polyols. For the PUR04 and PUR03 foams’ series, the compressive strength remained at the level of compressive strength of the reference foam up to 0.3 Eq of the eco-polyol. With a higher content of eco-polyols, it decreased to 107.89 kPa for PUR04.5 foam and 153.68 kPa for PUR03.5 foam, respectively. The reason for this was a significant increase in the content of less reactive polyols with a lower hydroxyl value and lower functionality than petrochemical polyol. This directly influenced the increase in apparent density and the decrease in compressive strength at the same time. The analysis of processing times (Table 6) showed that RPU foams with 0.4 and 0.5 Eq of eco-polyols had significantly extended string gel, tack free and free rise times. This meant that these foams could release a blowing agent from the inside because they did not have enough time to gelation foam structure. This would explain the twofold increase in apparent density and decrease in compressive strength due to the damaged internal structure of polyurethane [52,70].

Another dependence was noted for the apparent density and compressive strength of RPU/PIR foams. The increase in the amount of PLA0.4DEG and PLA0.3DEG eco-polyols resulted in a decrease in both parameters from 54.27 kg/m^3^ and 355.21 kPa for Ref. PIR foam to 42.62 kg/m^3^ and 275 kPa for PIR04.5 foam (0.5 Eq of PLA0.4DEG eco-polyol) and 40.89 kg/m^3^ and 240 kPa for PIR03.5 foam (0.5 Eq of PLA0.3DEG eco-polyol), respectively. The main reason for this dependence was the decrease in the degree of polyurethane cross-linking caused by the low hydroxyl value and the functionality of eco-polyols. Additionally, the linear structure of raw materials based on PLA waste increased the flexibility of the polyurethane matrix, while reducing its mechanical strength. This dependence was opposite to that obtained by Luo and Li. They carried out the glycolysis reaction of PET waste. Then, they used the obtained polyol for the synthesis of rigid polyurethane foams. They noted that an increase in the amount of polyol based on PET waste resulted in an increase in the apparent density and compressive strength of the foams. This was due to a higher hydroxyl value of recycling-based polyol and presence of aromatic rings in its structure (hard segments) [71].

**Figure 8:**
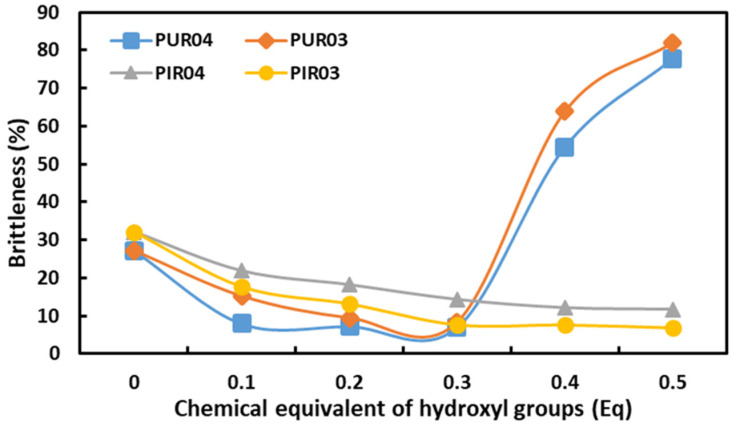
Dependence of brittleness on the content of eco-polyols based on PLA waste.

An important parameter of rigid polyurethane foams is also resistance to mechanical impacts. This parameter is best described by brittleness. The dependence between the brittleness of RPU and RPU/PIR foams and the content of eco-polyols is shown in Figure 8.

The new eco-polyols had a significant impact on the brittleness of RPU and RPU/PIR foams. The increase in the content of eco-polyols in RPU foams to 0.3 Eq and in RPU/PIR foams to 0.5 Eq resulted in a significant reduction in this parameter in comparison with the reference foams. For example, the brittleness of Ref. PUR foam was 27.18%, while after applying 0.3 Eq of eco-polyols it was 7.04% for PUR04.3 (0.3 Eq of PLA0.4DEG eco-polyol) and 8.3% for PUR03.3 (0.3 Eq of PLA0.3DEG eco-polyol), respectively. A further increase in the content of eco-polyols caused a significant increase in brittleness. This was due to the weakening of the structure of the polyurethane matrix caused by evaporation of the blowing agent before the foam gelled (as described earlier). In the case of RPU/PIR foams, the increase in the number of eco-polyols resulted in a reduction in brittleness from 32.14% for Ref. PIR foam to 11.75% for PIR04.5 foam and 6.92% for PIR03.5. The number of flexible segments in the polyurethane structure increased along with the increase in the content of eco-polyols in the formulation. This resulted in a decrease in its stiffness. It is known that polyester-based polyols have more elastic segments than polyether-based polyols. This is due to their chemical structure. Therefore, the addition of linear polyesterol in place of petrochemical polyetherol promotes a decrease in the stiffness of the obtained materials [9,72].

**Figure 9:**
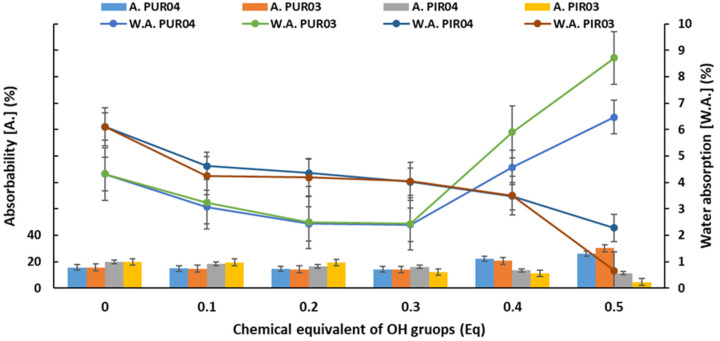
Dependence of absorbability (A.) and water absorption (W.A.) on the content of eco-polyols based on PLA waste.

Important application parameters of the porous materials are absorbability and water absorption. The first of these was the percentage amount of water in the material, immediately after removal from immersion. The second one was the percentage amount of water that stayed inside the composites. The dependence of absorbability and water absorption on the content of eco-polyols is shown in Figure 9.

RPU foams with the content of eco-polyols not higher than 0.3 Eq had lower absorbability and water absorption than the reference foam (A.—15.51% and W.A.—4.32%). These parameters were 14.13% and 2.40% for PUR04.3 (0.3 Eq of PLA0.4DEG eco-polyol) and 14.01% and 2.43% for PUR03.3 (0.3 Eq of PLA0.3DEG eco-polyol), respectively. A further increase in the content of eco-polyols in RPU foam formulations increased both parameters. This was a consequence of the structural changes that occurred during the synthesis of these materials. In the case of RPU/PIR foams, a gradual decrease in the values of absorbability and water absorption was noted along with an increase in the number of eco-polyols. The same dependencies were noted for the previous physicomechanical parameters. The decrease in the values of absorbability and water absorption of RPU/PIR foams was caused by the obtained closed-cell structure, which prevented the penetration of water into the foam [21,73]. Low water absorption capacity of polyurethane foams is an advantageous feature. It allows the use of these foams as insulating material in places with high humidity, without worrying about the decrease in insulation properties [74]. In contrast, the increase in absorbability and water absorption is an undesirable phenomenon, because it causes a decrease in the thermal insulation properties of PU foams [9,75].

#### 2.2.3. Aging Resistance Properties

Aging resistance tests of the obtained foams were carried out within 48 h, because the most characteristic changes occur in the first 24 to 72 h of their aging [11]. During the simulated aging of modified RPU/PIR foams, the determined changes in linear dimensions (ΔL) did not exceed ±1% and geometrical volume (ΔV) did not exceed ±3%. No close dependence was found between the values of these parameters and the number of eco-polyols. However, the share of eco-polyols in RPU/PIR foams had a positive effect on the aging resistance. The improvement of the stability of linear dimensions and geometric volume in comparison with the reference foam (ΔL = + 1.54% and ΔV = + 5.64%) was obtained. This was due to the introduction into the polyurethane matrix of long and linear polyester chains from PLA, which are relatively resistant to elevated temperatures. The flexible structure of such chains protects, for example, against deformation caused by migration of the blowing agent at elevated temperature [76]. The mass losses of reference foam and RPU/PIR foams modified by eco-polyols in all cases were below 4%. The value of this parameter was mainly related to the migration of the blowing agent contained inside the foam cells. A higher value of this parameter would suggest that the substances constituting the polyurethane matrix were decomposed [77].

Aging resistance tests of RPU foams showed that the tested foams are not resistant to high temperatures (120 °C). All samples subjected to this test, including the reference foam, were melted. It was impossible to determine changes in linear dimensions, geometric volume or mass loss at this temperature. It was experimentally determined that the maximum temperature at which it was possible to carry out this measurement was 70 °C. At this temperature, the change in linear dimensions of all foams was less than 1.5%, the change in geometric volume was less than 4%, and the mass loss was less than 2%. The difference in aging resistance of RPU and RPU/PIR foams resulted mainly from their chemical structure. RPU foams, unlike RPU/PIR foams, do not have isocyanurate rings in their structure, which significantly improve thermal resistance [78]. Moreover, as was mentioned earlier, lower values of HV and functionality of eco-polyols significantly reduced the degree of cross-linking of the obtained RPU foams. It also reduced the resistance to elevated temperature [79].

#### 2.2.4. Thermal Insulation Properties

**Figure 10:**
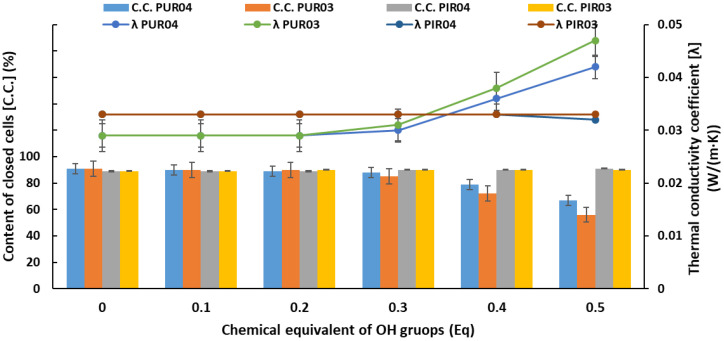
Dependence of the content of closed cells, the thermal conductivity coefficient and the content of eco-polyols based on PLA waste.

The most important property of rigid polyurethane foams for thermal insulation application is the value of the thermal conductivity coefficient λ. This parameter depends on the cell structure of the finished product, content of closed cells and type of blowing agent used [80,81]. The dependence between the λ coefficient, the content of closed cells and the content of eco-polyols is shown in Figure 10.

The thermal conductivity coefficient of the reference foams based on petrochemical polyol was 0.029 W/(m·K) for Ref. PUR and 0.033 W/(m·K) for Ref. PIR, respectively. The increase in the content of eco-polyols based on PLA wastes did not change this parameter in RPU/PIR foams. The λ value for all RPU/PIR foams modified by PLA0.4DEG and PLA0.3DEG polyols was 0.033 W/(m·K). However, significant changes were observed in the case of the modification of RPU foams. The increase in the content of eco-polyols resulted in an increase in the thermal conductivity coefficient. This increase was slight to 0.3 Eq of recycling-based polyol. The higher number of eco-polyols in the formulation caused a rapid increase in this parameter to 0.042 W/(m·K) for PUR04.5 foam and 0.047 W/(m·K) for PUR03.5, respectively. The reason for the changes in the value of the thermal conductivity coefficient is the change in the content of closed cells in the foam. The increase in the value of λ is due to the release of the blowing agent enclosed inside the foam cells. This means that the decrease in the content of closed cells improves the thermal conductivity of the material [7]. Such a dependence was noted in the case of RPU foams. The foams with the highest content of eco-polyols had the lowest values of closed cells: 67% (PUR04.5) and 56% (PUR03.5), respectively. The delayed gelation of polyurethane was the cause of structural changes, which significantly influenced all properties of these materials. In the case of RPU/PIR foams, the content of eco-polyols did not change the content of closed cells in the foam. For all foams, it was at the level of the reference foam (90%). Analyzing the results of research on thermal insulation properties of RPU foams up to 0.3 Eq of eco-polyols and all RPU/PIR foams, it was found that all λ values are within the industrial standard range for these types of materials. The value of the thermal conductivity coefficient of “water-blown” rigid polyurethane foams should be in the range 0.03–0.04 W/(m·K) [9]. However, in the case of replacing a petrochemical polyol by glycolysate or bio-polyol, the value of the λ coefficient may be in the range of 0.035–0.04 W/(m·K) [82,83].

#### 2.2.5. Structure of New Polyurethane Materials

**Figure 11:**
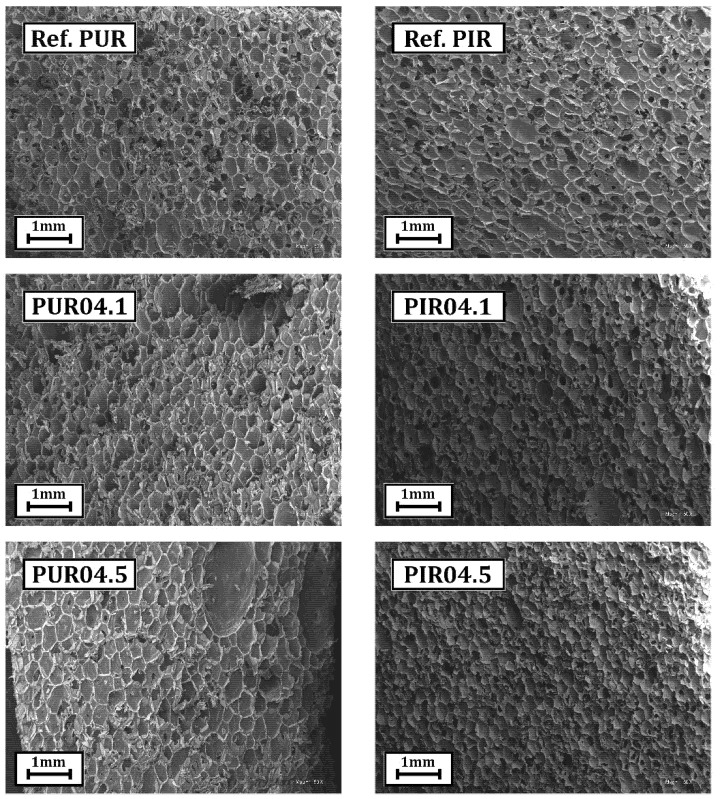

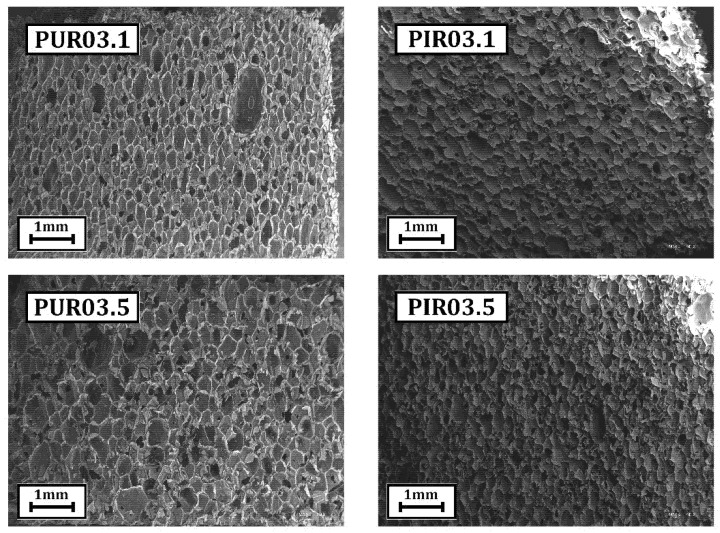
Micrographs of cellular structure of reference foams and foams modified by eco-polyols.

The cellular structure of foams is a very important parameter that determines practically all their properties, e.g., apparent density, compressive strength or thermal conductivity coefficient. The formation of an appropriate cellular structure (appropriate shape and size of pores) is influenced by the qualitative and quantitative composition of basic raw materials and additives used in the polyurethane formulation [9,11]. SEM micrographs of foams without eco-polyols, with their lowest content (0.1 Eq) and their highest content (0.5 Eq), were analyzed in order to verify the effect of new eco-polyols on the structure of rigid polyurethane and polyurethane/polyisocyanurate foams. The obtained micrographs are presented in Figure 11, and their statistical analysis is presented in Table 7.

The analysis of SEM micrographs of RPU foams for both eco-polyols showed that an increase in the glycolysate content caused even a 50% increase in the cell size. The larger cell diameters also increased the wall thickness of the foams from 14 µm for Ref. PUR foam to 19 µm for PUR04.5 foam and 17 µm for PUR03.5 foam. The increase in both parameters was mainly due to the decrease in polyurethane crosslinking resulting from the three-fold lower functionality of eco-polyols (Table 4) compared to the functionality of Rokopol RF-551 amounting to 4.5 and lower reactivity of eco-polyols. The lower reactivity delayed the gelation of the polyurethane, resulting in cells with larger diameters and thicker walls. Moreover, delayed gelation in relation to the evaporating blowing agent influenced the elongated shape of the cells. Correspondingly, lower cross-linking of the polyurethane matrix directly affected the deterioration of such parameters of RPU foams as compressive strength, brittleness or closed cell content [84]. On the other hand, the analysis of micrographs of RPU/PIR foams modified by eco-polyols showed an opposite dependence. A decrease in the average cell size and wall thickness was noted along with the increase in the content of eco-polyols. This dependence was due to the higher cross-linking density of the polyurethane matrix resulting from the presence of isocyanurate rings ensuring additional cross-linking of the polymer. Due to the presence of these groups (rings), the obtained foams retained their properties at the level of the reference foam. A similar dependence was observed by Czupryński et al. They used the product of glycolysis reaction of waste PU foam and DEG for synthesis RPU/PIR foams [82].

#### 2.2.6. Flammability Tests

**Figure 12:**
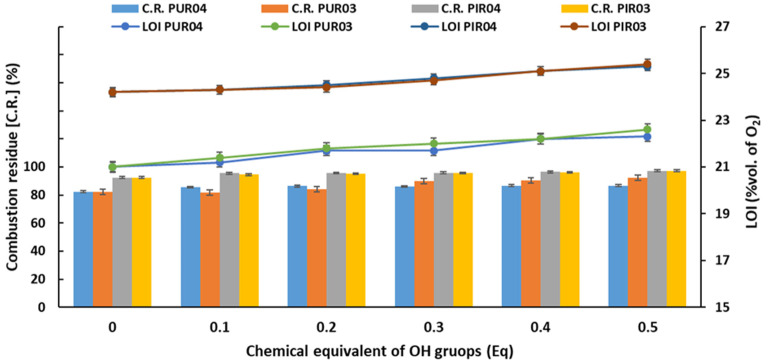
Dependence of the combustion residues (C.R.), limiting oxygen index (LOI) and the content of eco-polyols based on PLA waste.

The ease of ignition and release of large amounts of smoke during a fire, as well as the release of toxic gases, are significant difficulties associated with the use of PU porous materials. Increasing the flame retardancy of these materials is necessary to avoid safety issues and to comply with increasingly stringent legal requirements for plastics’ flammability. Safety issues necessitate the use of thermally stable and non-flammable materials, which release little heat and toxic gases [85]. Therefore, the newly obtained RPU and RPU/PIR foams were subjected to three flammability tests: Bütler’s combustion test, horizontal combustion test and limiting oxygen index (LOI). The dependence between LOI and combustion residue after vertical burning test and content of eco-polyols is shown in Figure 12.

Bütler’s vertical combustion test and the limited oxygen index (Figure 12) showed that the increased content of eco-polyols based on waste PLA caused a reduction in flammability. The combustion residue (C.R.) for RPU foams increased, respectively, from 82.36% for Ref. PUR to 86.63% for PUR04.5 (0.5 Eq of PLA0.4DEG eco-polyol) and to 92.23% for PUR03.5 (0.5 Eq of PLA0.3DEG eco-polyol). Additionally, the LOI of RPU foams increased from 21.0% for Ref. PUR to 22.3% for PUR04.5 and 22.6% for PUR03.5, respectively. The same dependence was noted for RPU/PIR foams. C.R. of RPU/PIR foams increased from 92.44% for Ref. PIR foam to 97.20% for PIR04.5 foam and 97.17% for PIR03.5 foam, while LOI increased from 24.2% for Ref. PIR to 25.3% for PIR04.5 and 25.4% for PIR03.5. The flame retardant effect did not result from the addition of eco-polyols into the foams but from the increased content of the flame retardant (Antiblaze TMCP) in the formulation. The increase in the amount of this additive resulted from the increase in the sum of the masses of polyol raw materials and polyisocyanate after addition of eco-polyols. This sum was the basis for calculation of the share of all additives in the RPU and RPU/PIR foams’ formulation. The horizontal combustion test classified these materials as self-extinguishing after withdrawal of the source of fire.

## 3. Materials and Methods

### 3.1. Raw Materials

**Figure 13:**
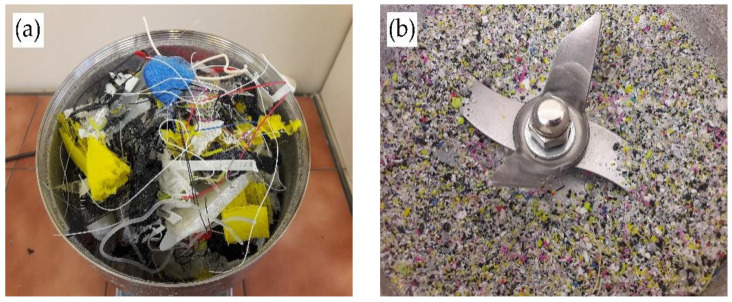
Appearance of PLA waste: (**a**) before grinding, (**b**) after grinding.

A mixture of polylactide waste was used for the synthesis of new eco-polyols. The waste used came from local PLA converters and 3D printing houses. In the used method [86], the source of PLA is irrelevant because it allows for the processing of polylactide from various sources, both natural and synthetic. Before synthesis, PLA was ground in a laboratory mill to a grain size below 5 mm. The appearance of the waste before and after grinding is shown in Figure 13.

Diethylene glycol at a concentration of 99% (Chempur, Piekary Śląskie, Poland) was also used for the synthesis of eco-polyols as a transesterifying agent of the bonds between lactic acid monomers. Solid anhydrous zinc stearate (produced by Chempur, Piekary Śląskie, Poland) was used as a reaction catalyst.

Eco-polyols based on PLA waste were used in the mixture with petrochemical polyol Rokopol RF-551—sorbitol oxyalkylation product, hydroxyl value of 420 mg KOH/g (produced by ZCh PCC Rokita SA, Brzeg Dolny, Poland) as a polyol component for synthesis of RPUFs and RPU/PIRFs. Purocyn B, a technical polyisocyanate (supplied by Purinova Ltd., Bydgoszcz, Poland) was used as the isocyanate raw material. The main component of it was 4,4′-diphenylmethane diisocyanate. The content of NCO groups was 31%.

The following additives were used for the preparation of RPU and RPU/PIR foams: 33% solution of DABCO (1,4-diazabicyclo[2,2,2]octane, produced by Alfa Aesar, Haverhill, MA, USA) in diethylene glycol (produced by Chempur, Piekary Śląskie, Poland), as a catalyst for urethane bond formation; Tegostab 8460 (polysiloxanepolyoxyalkylene surfactant, produced by Evonik, Essen, Germany), as a foam structure stabilizer; Antiblaze TMCP (trichloro-2-methylethyl phosphate, produced by Albemarle, Charlotte, NC, USA), as the flame retardant. Distilled water was used for production of blowing agent (carbon dioxide). It was produced in situ in reaction between distilled water and an excess of isocyanate raw material. In addition, for the preparation of RPU/PIR foams a 33% solution of anhydrous potassium acetate (produced by Chempur, Piekary Śląskie, Poland) in diethylene glycol (produced by Chempur, Piekary Śląskie, Poland) was also used, as a trimerization catalyst of NCO groups to isocyanurate rings.

### 3.2. Synthesis of New Eco-Polyols

**Figure 14:**
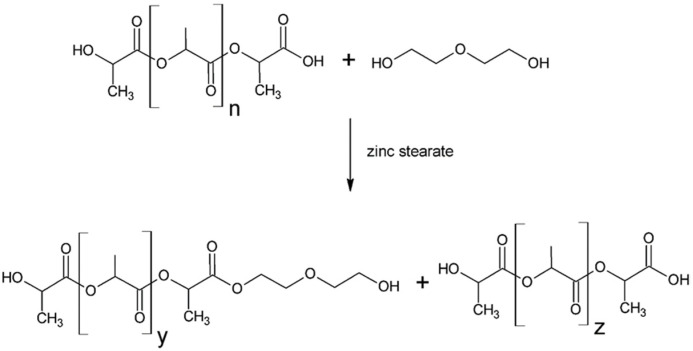
Transesterification reaction of PLA waste (n = y + z).

Diethylene glycol (DEG) at a concentration of 99% and anhydrous zinc stearate (ZS) were introduced into the reactor equipped with a mechanical stirrer, temperature sensor and reflux condenser. The obtained suspension was heated with continuous stirring (700 rpm) to 160 °C. Then, the ground PLA waste was introduced in small portions. The mass ratio of PLA:DEG:ZC reagents was 1:0.4:0.001 for PLA0.4DEG eco-polyol and 1:0.3:0.001 for PLA0.3DEG eco-polyol, respectively. After all the PLA waste had been added to the reactor, it was heated to 200 °C. The transesterification reaction of polylactide with diethylene glycol was carried out at this temperature (Figure 14). The reaction was carried out for approximately 3 h. The stabilization of the hydroxyl value at a constant level was assumed as the end of the reaction.

After the end of the reaction, the obtained eco-polyols were filtered hot under reduced pressure to remove the catalyst and any solid additives found in the PLA waste. The filtered polyols were cooled to ambient temperature and then tested to determine their suitability for the synthesis of polyurethane materials.

### 3.3. Examining the Properties of Eco-Polyols

Physicochemical, analytical and spectroscopic tests were performed on the new eco-polyols. This was for determining their suitability for the synthesis of RPUFs and RPU/PIRFs. Obtained polyol raw materials were also subjected to tests of susceptibility to biodegradation in soil environment.

#### 3.3.1. Physicochemical and Analytical Tests

The color and smell of obtained eco-polyols were tested organoleptically. The viscosity of the eco-polyols was determined using a Fungilab digital rheometer at 20 °C (293 K). The measurements were carried out using a standard spindle (DIN-87) working with the bushing (ULA-DIN-87). Maintenance of a constant temperature of measurement was provided by thermostat connected to the water jacket of the sleeve. Density was measured at 25 °C (298 K) in an adiabatic pycnometer in accordance with PN-EN 92/C-04504. The pH value was measured using a Hanna Instruments microprocessor laboratory pH-meter (ORP/ISO/°C) with RS22C connector.

The hydroxyl value (HV) and the acid value (AV) were determined in accordance with Purinova Ltd. standards—WT/06/07/PURINOVA. The water content (%_H2O_) was determined by the Karl Fischer method using a non-pyridine reagent of the trade name Titraqual in accordance with PN-81/C-04959. Elemental analysis of new compounds was carried out by Vario EL III CHNSO analyzer. The average molecular weights (M_n_) of the eco-polyols were determined by gel permeation chromatography (GPC) by using a Knauer chromatograph. The apparatus was equipped with thermostated columns and a refractometer detector. The measurements were made on the basis of calibration, by the use of polystyrene standards in the range of Mn from 162 to 25,500 g/mol. The functionality (f) was calculated on the basis of HV and average M_n_ of PLA-polyol.

#### 3.3.2. Spectroscopy Tests

The eco-polyols were tested in Fourier transform infrared (FTIR) spectroscopy by using a Nicolet iS20 spectrophotometer in a range from 400 to 4000 cm^−1^ and in nuclear magnetic resonance spectroscopy ^1^H NMR and ^13^C NMR using a Brücker NMR Ascend III spectrometer with a frequency of 400 MHz, in deuterated chloroform, as a solvent.

#### 3.3.3. Susceptibility to Biodegradation in Soil Environment

A biodegradation test of new eco-polyols was carried out in accordance with ISO 17556:2019 using the OxiTop Control S6 apparatus. It used a respirometric method to measure the oxygen demand necessary for aerobic biodegradation of polymeric materials in the soil environment. The measurement of consumed oxygen was presented using the value of biochemical oxygen demand (BOD), which is the number of milligrams of captured oxygen per mass unit of tested eco-polyol [49].

Sifted and dried garden soil with a high humus content and physicochemical parameters such as humidity of 5% (according to ISO 11274), pH of 6 (according to ISO 10390), grain diameter below 2 mm (collected in Szczepanowo, Kuyavian-Pomeranian Voivodeship, Poland) was used as a biodegradation environment. The measurement was carried out in a hermetic-closed system consisting of 200 mg of eco-polyol, 200 g of soil and 100 g of distilled water. It was placed in a laboratory incubator at 20 ± 0.2 °C and thermostated at this temperature for 28 days. The biochemical oxygen demand (BOD) for a single OxiTop Control S6 bottle was determined from Equation (2) taking into account the BOD of the tested system reduced by the BOD of the soil (reference sample) and concentration of the tested compound in the soil [49,66].

(2) $$BOD_{S}=\frac{BOD_{x}-BOD_{g}}{c}$$

where: *S*—number of measurement days, *BOD_S_*—biochemical oxygen demand of the analyzed sample within *S* days (mg/L), *BOD_x_*—biochemical oxygen demand of the measuring system (bottle with sample and soil) (mg/L), *BOD_g_*—biochemical oxygen demand of soil without a sample (mg/L), *c*—sample concentration in the tested system (mg/L).

The degree of biodegradation of eco-polyols was determined based on Equation (3):

(3) $$D_{t}=\frac{BOD_{S}}{TOD}\cdot 100\%$$

where: *D_t_*—degree of eco-polyol biodegradation (%), *TOD*—theoretical oxygen demand (mg/L). The theoretical oxygen demand for each system was calculated from Equation (4):

(4) $$TOD=\frac{16\cdot \left(2c+0.5h-o\right)}{m}$$

where: *c*, *h*, *o*—mass shares of carbon, hydrogen and oxygen in the molecule of biodegradable material (-), *m*—weight of biodegradable material (*g*).

### 3.4. Preparation of RPU and RPU/PIR Foams

Formulation of rigid polyurethane and polyurethane/polyisocyanurate foams with eco-polyols based on PLA waste required an experimental investigation to determine the optimal composition of additives (catalysts, surfactant, flame retardant and blowing agent). The hydroxyl value was the basis for determining the amount of polyol raw materials. These values allowed us to calculate the mass equivalent (Eq_OH_) of the petrochemical polyol and eco-polyol. The addition of isocyanate raw material was selected taking into account equivalent ratio of NCO to OH groups (Eq_NCO_:Eq_OH_) in the reaction mixture. For RPU foams, this ratio was 1.7:1, while for RPU/PIR foams it was 3.7:1. An excess of isocyanate raw material was necessary for reaction between NCO and OH groups to produce a urethane bond, reaction between NCO group and distilled water to produce a blowing agent (carbon dioxide) and trimerization reaction of three NCO groups to produce an isocyanurate ring in RPU/PIR foams. The sum of mass equivalents of petrochemical polyol and eco-polyol was always 1.

The content of additives for RPU foams (in weight percentage to sum of polyols masses) was: urethane bond catalyst—3 wt.%, flame retardant—30 wt.%, and surfactant—1.7 wt.%. Content of additives for RPU/PIR foams (in weight percentage to sum of polyols and polyisocyanate masses) was: urethane bond catalyst—1 wt.%, isocyanate trimerization catalyst—2.5 wt.%, flame retardant—17 wt.%, and surfactant—1.7 wt.%. Distilled water was always added in an amount of 0.7 EqOH for synthesis of RPU and RPU/PIR foams. This means that each time the calculated amount of water was reduced by the amount of water added with eco-polyols.

Foams were obtained at a laboratory scale using the one-stage method, from the two-component system—A and B. Component A was obtained as a result of mixing of appropriate amounts of petrochemical polyol, eco-polyol, catalysts, surfactant, blowing agent and flame retardant. Component B was Purocyn B. Components A and B were mixed for 10 s with a mechanical stirrer (1800 rpm) in a suitable mass ratio. The mixture was poured into a cuboidal mold with internal dimensions of 25 cm × 25 cm × 30 cm, where the growth of foam proceeded freely. The synthesis of foams was twice repeated. Obtained polyurethane materials were thermostated for six hours at 120 °C in a laboratory dryer with forced circulation, after removal from the mold. The formulation of RPU and RPU/PIR foams modified by PLA0.4DEG and PLA0.3DEG polyols are shown in Table 8, Table 9, Table 10 and Table 11.

Reference foams and five types of each foam were obtained. Modified foams were obtained by replacing petrochemical polyol (Rokopol RF-551) with polyols based on PLA waste. The obtained foams were cut into standardized samples and subjected to further testing.

### 3.5. Selected Properties of RPU and RPU/PIR Foams

#### 3.5.1. Foaming Process

The foaming process was analyzed by electronic stopwatch to determine the characteristic foaming times in accordance with ASTM D7487 13e^1^. Cream, free rise, string gel and tack free times were measured during synthesis of the RPU and RPU/PIR foams.

#### 3.5.2. Physicomechanical Properties of New Polyurethane Materials

New RPU and RPU/PIR foams have been tested for: apparent density in accordance with ISO 845:2006, compressive strength on a universal strength machine Instron 5544 in accordance with ISO 844:2014, brittleness in accordance with ASTM C-421-61, absorbability and water absorption in accordance with ISO 2896:2001.

#### 3.5.3. Aging Resistance Properties

Aging resistance of the obtained foams was carried out in a thermostating process of cubic samples with a side length of 50 mm for 48 h at a temperature of 120 °C. The result of this test included a change of linear dimensions (ΔL), change of geometrical volume (ΔV) and mass loss (Δm). The values of these parameters were calculated in accordance with ISO 1923:1981 and ISO 4590:2016–11.

#### 3.5.4. Thermal Insulation Properties

Thermal conductivity of the foams was determined based on the determination of the thermal conductivity coefficient λ in accordance with ISO 8301:1991. Tests were carried out with the FOX 200 apparatus, in the measurement range of λ equal to 20–100 mW/(m·K). Measurements were performed in the series at intervals of 0.5 s and at an average measuring temperature of 10 °C (temperature of hot plate—20 °C, temperature of cold plate—0 °C).

The content of closed cells was determined in accordance with ISO 4590:2016-11 using the helium pycnometer AccuPyc 1340 with the FoamPyc. This software calculated the content of closed cells based on the measurement of pressure changes in the test chamber.

#### 3.5.5. Structure of New Polyurethane Materials

Microstructure of cells was analyzed by scanning electron microscope (SEM) HITACHI SU8010. The foam samples were cut with a scalpel in order to obtain a uniform thickness of the samples with an undamaged structure before the test. Then, a gold layer was sprayed on them. The tests were carried out at the accelerating voltage of 30 kV, with the working distance of 10 mm and magnification of 50×. Statistical analysis of cell sizes, wall thickness and content of cells per area unit were carried out on the basis of obtained micrographs using ImageJ software.

#### 3.5.6. Flammability Tests

Three flammability tests were performed on the new RPU and RPU/PIR foams: Bütler’s combustion test (vertical test) in accordance with ASTM D3014-73, horizontal combustion test in accordance with PN-78 C-05012, and limiting oxygen index (LOI) using Concept Equipment apparatus in accordance with ASTM D 2863-1970.

## 4. Conclusions

As part of this research, the glycolysis reaction of PLA waste was used as a method of chemical recycling of this polymer. Diethylene glycol (DEG) in the presence of an organometallic catalyst was used as a glycolyzing agent. The results of the research work were two new eco-polyols obtained at different mass ratios of PLA to DEG of 1:0.4 and 1:0.3, respectively. The obtained compounds were subjected to detailed physicochemical, analytical and spectroscopic tests. The susceptibility to biodegradation of the new eco-polyols under controlled soil environment conditions was also tested. The obtained recycling-based polyols were odorless liquids with comparable values of density (approx. 1.24 g/cm^3^) and pH (approx. 6.6) and viscosity values depending on the used mass ratio of reagents. The reduction in the amount of DEG in the reaction mixture resulted in a four-fold increase in viscosity. It was also observed that the DEG content influenced the hydroxyl value and the functionality of the new eco-polyols. Changing the mass ratio of PLA:DEG from 1:0.4 to 1:0.3 resulted in a 20% reduction in the hydroxyl value from 261.64 mg KOH/g to 209.87 mg KOH/g. The ratio of the reactants did not affect the functionality of the polyol raw materials, which was approximately 1.55. The acid number of the compounds obtained was about 2 mg KOH/g, and the water content was about 0.2 wt.%. Spectroscopic tests confirmed the assumed chemical structure of the obtained compounds, confirming their suitability for the synthesis of polyurethane materials.

New eco-polyols were used to synthesize rigid polyurethane and polyurethane-polyisocyanurate foams. The content of new compounds in the obtained materials was increased from 0 to 0.5 of chemical equivalents of hydroxyl groups, replacing petrochemical polyol with them. The obtained foams were tested for, among others, physical and mechanical properties or thermal insulation properties. It was noted that the use of eco-polyols had different effects on RPU and RPU/PIR foams. In the case of rigid polyurethane foams, improvement in performance properties up to 0.3 Eq of eco-polyols has been noted (e.g., lower brittleness or higher compressive strength). However, the higher amount of these polyols led to the deterioration of all the properties of RPU foams. It was different in the case of RPU/PIR foams. The use of eco-polyols based on DEG and PLA waste improved most of the properties, e.g., reduced the apparent density from 54 kg/m^3^ to 42 kg/m^3^ and brittleness from 32% to 7%, improved aging resistance or retained thermal conductivity at the level of the reference foam (0.033 W/(m·K)).

The origin, chemical structure and properties of the obtained eco-polyols indicate that they can be an ecological alternative to petrochemical raw materials used in the polyurethane industry. This is also confirmed by the obtained results of the RPU and RPU/PIR foam tests. Due to their origin, method of obtainment and properties, they perfectly represent a sustainable model of the circular economy. They are an example of an innovative product for traditional, non-renewable raw materials of petrochemical origin and allow for efficient use of the existing resources. Moreover, they fit perfectly with the doctrine of sustainable development and the principles of green chemistry.

## Figures and Tables

**Table 1:** Physicochemical properties of new eco-polyols based on waste PLA.

Parameter	PLA0.4DEG	PLA0.3DEG
Color (-)	black	black
Smell (-)	odorless	odorless
Density (g/cm^3^)	1.24 ± 0.01	1.24 ± 0.02
Viscosity (mPa·s)	2459 ± 96	8681 ± 342
pH (-)	6.6 ± 0.1	6.6 ± 0.1

**Table 2:** Analytical test results of new eco-polyols based on waste PLA.

Parameter	PLA0.4DEG	PLA0.3DEG
HV (mg KOH/g)	261.64 ± 3.25	209.87 ± 2.87
AV (mg KOH/g)	1.93 ± 0.12	2.05 ± 0.14
%_H2O_ (%wt.)	0.10 ± 0.01	0.25 ± 0.01

**Table 3:** Results of elemental analysis of new eco-polyols based on waste PLA.

Element	Carbon (%)	Hydrogen (%)	Oxygen (%)
PLA0.4DEG	41.47 ± 0.13	7.94 ± 0.15	50.59 ± 0.18
PLA0.3DEG	41.15 ± 0.11	7.70 ± 0.08	51.15 ± 0.15

**Table 4:** Results of GPC analysis and functionality of eco-polyols based on PLA waste.

Parameter	M_n_ (g/mol)	M_w_ (g/mol)	D (-)	f (-)
PLA0.4DEG	341	401	1.18	1.59
PLA0.3DEG	414	529	1.27	1.55

**Table 5:** Results of susceptibility to biodegradation of eco-polyols based on waste PLA.

Parameter	BOD_28_ (mg/L)	TOD (mg/L)	Dt (%)
PLA0.4DEG	154.5	26.41	100 *
PLA0.3DEG	159.5	25.45	100 *

* a result higher than 100% means complete biodegradation of the tested material [49].

**Table 6:** Processing times for RPU foams based on eco-polyols from waste PLA.

Sample	Cream Time(s)	String Gel Time (s)	Tack Free Time (s)	Free Rise Time (s)
Ref. PUR	17 ± 1	54 ± 1	84 ± 1	75 ± 1
PUR04.1	17 ± 1	56 ± 1	85 ± 1	77 ± 1
PUR04.2	17 ± 1	60 ± 1	85 ± 1	78 ± 1
PUR04.3	18 ± 1	72 ± 1	96 ± 1	89 ± 1
PUR04.4	21 ± 1	102 ± 1	123 ± 2	115 ± 1
PUR04.5	22 ± 1	115 ± 2	156 ± 2	134 ± 2
PUR03.1	18 ± 1	102 ± 2	155 ± 2	119 ± 1
PUR03.2	19 ± 1	125 ± 2	175 ± 2	142 ± 2
PUR03.3	20 ± 1	139 ± 2	199 ± 2	162 ± 2
PUR03.4	23 ± 1	275 ± 2	457 ± 5	296 ± 2
PUR03.5	24 ± 1	326 ± 2	560 ± 6	372 ± 3

**Table 7:** Results of SEM micrographs’ analysis.

Sample	Cell Size (μm)	Thickness of Cell Wall (μm)	Content of Cells per Area Unit (cells/mm^2^)
Ref. PUR	332 ± 41	14 ± 3	11 ± 3
PUR04.1	344 ± 51	14 ± 3	9 ± 3
PUR04.5	517 ± 102	19 ± 3	6 ± 3
PUR03.1	339 ± 62	14 ± 3	11 ± 2
PUR03.5	441 ± 73	17 ± 2	7 ± 2
Ref. PIR	391 ± 52	15 ± 3	8 ± 3
PIR04.1	373 ± 44	13 ± 3	10 ± 2
PIR04.5	296 ± 39	13 ± 3	12 ± 2
PIR03.1	356 ± 34	14 ± 2	10 ± 3
PIR03.5	304 ± 43	12 ± 3	12 ± 2

**Table 8:** Formulation of RPU foams with PLA0.4DEG eco-polyol.

Sample	Rokopol RF-551 (Eq_OH_) (g)	PLA0.4DEG (Eq_OH_) (g)	Tegostab 8460 (g)	33% DABCO (g)	Antiblaze TMCP (g)	Distilled Water (g)	Purocyn B (Eq_NCO_) (g)
Ref. PUR	*1.0* 100.00	*0.0* 0.00	1.70	3.00	30.00	4.73	*1.7* 172.71
PUR04.1	*0.9* 90.00	*0.1* 16.08	1.80	3.18	31.82	4.71	*1.7* 172.71
PUR04.2	*0.8* 80.00	*0.2* 32.16	1.91	3.36	33.65	4.69	*1.7* 172.71
PUR04.3	*0.7* 70.00	*0.3* 48.24	2.01	3.55	35.47	4.68	*1.7* 172.71
PUR04.4	*0.6* 60.00	*0.4* 64.33	2.11	3.73	37.30	4.66	*1.7* 172.71
PUR04.5	*0.5* 50.00	*0.5* 80.41	2.22	3.91	39.12	4.64	*1.7* 172.71

**Table 9:** Formulation of RPU/PIR foams with PLA0.4DEG eco-polyol.

Sample	Rokopol RF-551 (Eq_OH_) (g)	PLA0.4DEG (Eq_OH_) (g)	Tegostab 8460 (g)	33% DABCO (g)	33% Potassium Acetate (g)	Antiblaze TMCP (g)	Distilled Water (g)	Purocyn B (Eq_NCO_) (g)
Ref. PIR	*1.0* 66.80	*0.0* 0.00	5.40	3.17	7.93	53.96	3.17	*3.7* 250.60
PIR04.1	*0.9* 60.12	*0.1* 10.72	5.46	3.21	8.04	54.64	3.16	*3.7* 250.60
PIR04.2	*0.8* 53.44	*0.2* 21.44	5.53	3.25	8.14	55.33	3.15	*3.7* 250.60
PIR04.3	*0.7* 46.76	*0.3* 32.16	5.60	3.30	8.24	56.02	3.14	*3.7* 250.60
PIR04.4	*0.6* 40.07	*0.4* 42.88	5.67	3.34	8.34	56.70	3.13	*3.7* 250.60
PIR04.5	*0.5* 33.39	*0.5* 53.60	5.74	3.38	8.44	57.39	3.12	*3.7* 250.60

**Table 10:** Formulation of RPU foams with PLA0.3DEG eco-polyol.

Sample	Rokopol RF-551 (Eq_OH_) (g)	PLA0.3DEG (Eq_OH_) (g)	Tegostab 8460 (g)	33% DABCO (g)	Antiblaze TMCP (g)	Distilled Water (g)	Purocyn B (Eq_NCO_) (g)
Ref. PUR	*1.0* 100.00	*0.0* 0.00	1.70	3.00	30.00	4.73	*1.7* 172.71
PUR03.1	*0.9* 90.00	*0.1* 20.05	1.87	3.30	33.01	4.67	*1.7* 172.71
PUR03.2	*0.8* 80.00	*0.2* 40.10	2.04	3.60	36.03	4.62	*1.7* 172.71
PUR03.3	*0.7* 70.00	*0.3* 60.14	2.21	3.90	39.04	4.57	*1.7* 172.71
PUR03.4	*0.6* 60.00	*0.4* 80.19	2.38	4.21	42.06	4.52	*1.7* 172.71
PUR03.5	*0.5* 50.00	*0.5* 100.24	2.55	4.51	45.07	4.47	*1.7* 172.71

**Table 11:** Formulation of RPU/PIR foams with PLA0.3DEG eco-polyol.

Sample	Rokopol RF-551 (Eq_OH_) (g)	PLA0.3DEG (Eq_OH_) (g)	Tegostab 8460 (g)	33% DABCO (g)	33% Potassium Acetate (g)	Antiblaze TMCP (g)	Distilled Water (g)	Purocyn B (Eq_NCO_) (g)
Ref. PIR	*1.0* 66.80	*0.0* 0.00	5.40	3.17	7.93	53.96	3.17	*3.7* 250.60
PIR03.1	*0.9* 60.12	*0.1* 13.37	5.51	3.24	8.10	55.09	3.14	*3.7* 250.60
PIR03.2	*0.8* 53.44	*0.2* 26.73	5.62	3.31	8.27	56.23	3.10	*3.7* 250.60
PIR03.3	*0.7* 46.76	*0.3* 40.10	5.74	3.37	8.44	57.37	3.07	*3.7* 250.60
PIR03.4	*0.6* 40.07	*0.4* 53.46	5.85	3.44	8.60	58.50	3.04	*3.7* 250.60
PIR03.5	*0.5* 33.39	*0.5* 66.83	5.96	3.51	8.77	59.64	3.00	*3.7* 250.60

## Data Availability

Data is contained within the article.
